# Supplementary material for: Multi‐dataset identification of innovative feature genes and molecular mechanisms in keratoconus
Source: J Cell Mol Med. 2024 Sep 19;28(18):e70079. doi: 10.1111/jcmm.70079 (PMC11412914; doi:10.1111/jcmm.70079)
Supplement: Supplementary file 2 — Material S2. [file JCMM-28-e70079-s002.zip › 7.GSEA analysis/6.SNORA74B/KEGG_GLYCOSYLPHOSPHATIDYLINOSITOL_GPI_ANCHOR_BIOSYNTHESIS.html]

Details for gene set KEGG\_GLYCOSYLPHOSPHATIDYLINOSITOL\_GPI\_ANCHOR\_BIOSYNTHESIS[GSEA]

|  || Dataset | SNORA74B.group.cls#h\_versus\_l |
| Phenotype | group.cls#h\_versus\_l |
| Upregulated in class | h |
| GeneSet | KEGG\_GLYCOSYLPHOSPHATIDYLINOSITOL\_GPI\_ANCHOR\_BIOSYNTHESIS |
| Enrichment Score (ES) | 0.24239272 |
| Normalized Enrichment Score (NES) | 0.75034815 |
| Nominal p-value | 0.7046843 |
| FDR q-value | 1.0 |
| FWER p-Value | 1.0 |
Table: GSEA Results Summary

  

Fig 1: Enrichment plot: KEGG\_GLYCOSYLPHOSPHATIDYLINOSITOL\_GPI\_ANCHOR\_BIOSYNTHESIS      
 Profile of the Running ES Score & Positions of GeneSet Members on the Rank Ordered List

  

| SYMBOL | TITLE | RANK IN GENE LIST | RANK METRIC SCORE | RUNNING ES | CORE ENRICHMENT || 1 | PGAP1 | na | 3175 | 0.292 | 0.0681 | Yes |
| 2 | PIGL | na | 5810 | 0.199 | 0.1007 | Yes |
| 3 | PIGY | na | 12826 | 0.049 | -0.0746 | Yes |
| 4 | PIGM | na | 12950 | 0.047 | -0.0522 | Yes |
| 5 | PIGW | na | 15660 | 0.018 | -0.1205 | Yes |
| 6 | GPAA1 | na | 17270 | 0.002 | -0.1658 | Yes |
| 7 | GPLD1 | na | 17677 | -0.002 | -0.1762 | Yes |
| 8 | PIGG | na | 19404 | -0.022 | -0.2139 | Yes |
| 9 | PIGN | na | 19665 | -0.025 | -0.2078 | Yes |
| 10 | PIGS | na | 19929 | -0.028 | -0.2003 | Yes |
| 11 | PIGP | na | 20476 | -0.034 | -0.1976 | Yes |
| 12 | PIGX | na | 21204 | -0.042 | -0.1953 | Yes |
| 13 | PIGK | na | 21882 | -0.050 | -0.1874 | Yes |
| 14 | PIGH | na | 22296 | -0.055 | -0.1690 | Yes |
| 15 | PIGF | na | 22309 | -0.056 | -0.1390 | Yes |
| 16 | PIGO | na | 22350 | -0.056 | -0.1095 | Yes |
| 17 | PIGT | na | 23624 | -0.075 | -0.1052 | Yes |
| 18 | DPM2 | na | 23681 | -0.076 | -0.0654 | Yes |
| 19 | PIGU | na | 23941 | -0.080 | -0.0292 | Yes |
| 20 | PIGQ | na | 24502 | -0.089 | 0.0035 | Yes |
| 21 | PIGV | na | 24704 | -0.093 | 0.0485 | Yes |
| 22 | PIGC | na | 24928 | -0.097 | 0.0952 | Yes |
| 23 | PIGA | na | 25429 | -0.106 | 0.1386 | Yes |
| 24 | PIGZ | na | 25832 | -0.113 | 0.1889 | Yes |
| 25 | PIGB | na | 26316 | -0.123 | 0.2424 | Yes |
Table: GSEA details [plain text format]

  

Fig 2: KEGG\_GLYCOSYLPHOSPHATIDYLINOSITOL\_GPI\_ANCHOR\_BIOSYNTHESIS      
 Blue-Pink O' Gram in the Space of the Analyzed GeneSet

  

Fig 3: KEGG\_GLYCOSYLPHOSPHATIDYLINOSITOL\_GPI\_ANCHOR\_BIOSYNTHESIS: Random ES distribution      
 Gene set null distribution of ES for **KEGG\_GLYCOSYLPHOSPHATIDYLINOSITOL\_GPI\_ANCHOR\_BIOSYNTHESIS**
